# Effect of metformin versus placebo on metabolic factors in the MA.32 randomized breast cancer trial

**DOI:** 10.1038/s41523-021-00275-z

**Published:** 2021-06-08

**Authors:** Pamela J. Goodwin, Ryan J. O. Dowling, Marguerite Ennis, Bingshu E. Chen, Wendy R. Parulekar, Lois E. Shepherd, Margot J. Burnell, Rachel Vander Meer, Andrea Molckovsky, Anagha Gurjal, Karen A. Gelmon, Jennifer A. Ligibel, Dawn L. Hershman, Ingrid A. Mayer, Timothy J. Whelan, Timothy J. Hobday, Priya Rastogi, Manuela Rabaglio-Poretti, Julie Lemieux, Alastair M. Thompson, Daniel W. Rea, Vuk Stambolic

**Affiliations:** 1 Lunenfeld-Tanenbaum Research Institute, Mount Sinai Hospital, and Department of Medicine, University of Toronto, Toronto, ON Canada; 2 Hoffman-La Roche Limited, Mississauga, ON Canada; 3 Applied Statistician, Markham, ON Canada; 4 Canadian Cancer Trials Group, Queen’s University, Kingston, ON Canada; 5 Department of Oncology, Saint John Regional Hospital, St. John, NB Canada; 6 Department of Oncology, Niagara Health System, St. Catharines, ON Canada; 7 Department of Medical Oncology, Grand River Regional Cancer Centre, Kitchener, ON Canada; 8 Abbotsford Centre, British Columbia Cancer Agency, Abbotsford, BC Canada; 9 University of British Columbia, BC Cancer Agency, Vancouver, BC Canada; 10 Dana-Farber Cancer Institute, Boston, MA USA; 11 Herbert Irving Comprehensive Cancer Center, Columbia University Medical Center, Columbia, NY USA; 12 Vanderbilt University, Nashville, TN USA; 13 McMaster University, Juravinski Cancer Centre, Hamilton, ON Canada; 14 Mayo Clinic, Rochester, MN USA; 15 NRG Oncology and University of Pittsburgh Medical Center, Pittsburgh, PA USA; 16 IBCSG and Department of Oncology, Bern University Hospital, University of Bern, Berne, Switzerland; 17 CHU de Québec-Université Laval, Québec, QC Canada; 18 Baylor College of Medicine, Houston, TX USA; 19 Cancer Research UK Clinical Trials Unit (CRCTU), Institute of Cancer and Genomic Sciences, University of Birmingham, Birmingham, UK; 20 Princess Margaret Cancer Centre, University Health Network, Toronto, ON Canada; 21 Department of Medical Biophysics, University of Toronto, Toronto, ON Canada

**Keywords:** Translational research, Breast cancer, Cancer metabolism

## Abstract

Metformin may exert anticancer effects through indirect (mediated by metabolic changes) or direct mechanisms. The goal was to examine metformin impact on metabolic factors in non-diabetic subjects and determine whether this impact varies by baseline BMI, insulin, and rs11212617 SNP in CCTG MA.32, a double-blind placebo-controlled randomized adjuvant breast cancer (BC) trial. 3649 subjects with T1-3, N0-3, M0 BC were randomized; pretreatment and 6-month on-treatment fasting plasma was centrally assayed for insulin, leptin, highly sensitive C-reactive protein (hsCRP). Glucose was measured locally and homeostasis model assessment (HOMA) calculated. Genomic DNA was analyzed for the rs11212617 SNP. Absolute and relative change of metabolic factors (metformin versus placebo) were compared using Wilcoxon rank and *t*-tests. Regression models were adjusted for baseline differences and assessed interactions with baseline BMI, insulin, and the SNP. Mean age was 52 years. The majority had T2/3, node positive, hormone receptor positive, HER2 negative BC treated with (neo)adjuvant chemotherapy and hormone therapy. Median baseline body mass index (BMI) was 27.4 kg/m^2^ (metformin) and 27.3 kg/m^2^ (placebo). Median weight change was −1.4 kg (metformin) vs +0.5 kg (placebo). Significant improvements were seen in all metabolic factors, with 6 month standardized ratios (metformin/placebo) of 0.85 (insulin), 0.83 (HOMA), 0.80 (leptin), and 0.84 (hsCRP), with no qualitative interactions with baseline BMI or insulin. Changes did not differ by rs11212617 allele. Metformin (vs placebo) led to significant improvements in weight and metabolic factors; these changes did not differ by rs11212617 allele status.

## Introduction

The anti-diabetes drug metformin has been associated with reduced cancer risk and improved cancer prognosis in observational studies^1,2^; it may exert beneficial effects on cancer through both indirect and direct mechanisms. Indirect effects are likely mediated by systemic reductions in insulin, which results in decreased tumor-specific activation of the insulin receptor (IR) and suppression of mitogenic PI3K and Ras signaling^3^. Alternatively, metformin may exhibit direct inhibitory effects on cancer cells that are achieved by liver kinase B1 (LKB1)-mediated activation of AMP-activated protein kinase (AMPK), a negative regulator of PI3K/Akt/mTOR signaling and protein synthesis^4^. While several clinical intervention studies have explored possible anticancer effects of metformin^5–9^, none has assessed impact on patient outcome in the adjuvant setting. Canadian Cancer Trials Group (CCTG) MA.32 was initiated in 2010 to test the effect of metformin (versus placebo) on breast cancer (BC) outcomes in women with high-risk early-stage BC cancer who were receiving standard therapy.

We have previously reported^10^ improvements in body weight and circulating blood factors [glucose, insulin, leptin, highly sensitive C-reactive protein (hsCRP), homeostasis model assessment (HOMA)] in a preplanned safety analysis of the first 492 MA.32 subjects. Here, we report an in-depth analysis of the effect of metformin versus placebo on change in body weight and circulating metabolic factors in the full MA.32 study population, exploring interactions with baseline BMI and insulin. In addition, we examine the impact of the rs11212617 single-nucleotide polymorphism (SNP) located near the ATM gene on these variables. Glycemic response to metformin has been linked to this SNP in some, but not all, studies^11–14^. Specifically, presence of the minor allele (C) in diabetic patients has been associated with greater reduction in glycated hemoglobin (HbA1C). The status of this SNP has also been associated with metformin benefit in tumor response in human epidermal growth factor receptor 2 (HER2) positive BC^15^.

## Results

### Study population

**Fig. 1:**
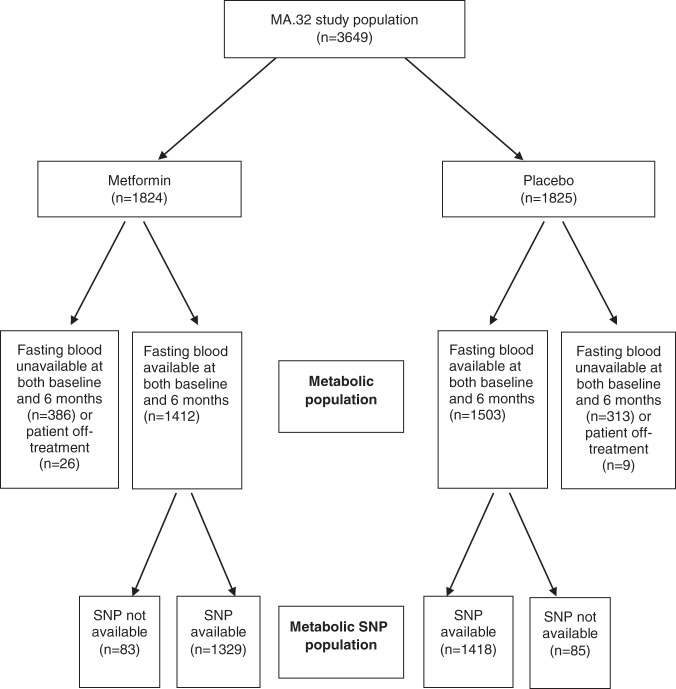
CONSORT diagram—identification of subjects included in the metabolic and metabolic SNP populations from the full MA.32 study population. SNP single-nucleotide polymorphism.

3649 women were randomized (1824 metformin, 1825 placebo—see Fig. 1). Fasting blood was available at both baseline and on-treatment at 6 months in 1412 metformin and 1503 placebo subjects; these subjects represent the metabolic population in this paper. SNP information was available on 1329 of the metformin patients and 1418 of the placebo patients—these patients represent the metabolic SNP population.

Baseline characteristics of the subjects included in, and excluded from, the metabolic analyses are shown in Table 1. It can be seen that subjects included (vs excluded) in the metabolic analyses were less likely to have been randomized to metformin (48.4% included vs 56.1% excluded) than placebo (51.6 vs 43.9, *p* < 0.001 compared to metformin), less likely to be African American (3.8% vs 7.5%, *p* < 0.001), more likely to have had hormone receptor-positive breast cancer (70.5% vs 65%, *p* = 0.004) and to have received hormone therapy (62.7% vs 56.1%, *p* = 0.003). They were less likely to have received neoadjuvant chemotherapy (19.7% vs 26.3%, *p* < 0.001); receipt of any adjuvant chemotherapy was similar in included and excluded subjects (89.3% vs 88.8%). The rs11212617 SNP genotyping was performed on 94.2% of the patients in the metabolic population.

**Table 1:** Baseline patient and tumor characteristics.

	Included and excluded from metabolic population			Metabolic population study arms	
	Included	Excluded	*P*	Metformin	Placebo
	*N* = 2915	N = 734		*N* = 1412	*N* = 1503
Treatment arm			<0.001		
Metformin	1412 (48.4%)	412 (56.1%)			
Placebo	1503 (51.6%)	322 (43.9%)			
Age (years)			0.86		
Mean (SD)	52.4 (10.0)	52.3 (10.5)		52.0 (10.0)	52.7 (10.0)
BMI (kg/m^2^)			0.35		
<25	952 (32.7%)	222 (30.2%)		460 (32.6%)	492 (32.7%)
≥25 and <30	963 (33.0%)	242 (33%)		460 (32.6%)	503 (33.5%)
≥30	1000 (34.3%)	270 (36.8%)		492 (34.8%)	508 (33.8%)
Race			<0.001		
Asian	78 (2.7%)	21 (2.9%)		35 (2.5%)	43 (2.9%)
Black or African American	112 (3.8%)	55 (7.5%)		54 (3.8%)	58 (3.9%)
American Indian or Alaska Native or Native Hawaiian or Pacific Islander	26 (0.9%)	4 (0.5%)		13 (0.9%)	13 (0.9%)
White	2667 (91.5%)	637 (86.8%)		1295 (91.7%)	1372 (91.3%)
Not reported/refused to answer	32 (1.1%)	17 (2.3%)		15 (1.1%)	17 (1.1%)
T stage (any neoadjuvant)			0.294		
cT1a + cT1b + cT1c	57 (9.9%)	27 (13.9%)		18 (6.7%)	39 (12.6%)
cT2	329 (57%)	105 (54.1%)		162 (60.7%)	167 (53.9%)
cT3	191 (33.1%)	62 (32%)		87 (32.6%)	104 (33.5%)
T stage (no neoadjuvant)			0.187		
pT1a + pT1b + pT1c + pT1mic	926 (39.6%)	229 (42.4%)		443 (38.7%)	483 (40.5%)
pT2	1227 (52.5%)	282 (52.2%)		605 (52.8%)	622 (52.1%)
pT3	184 (7.9%)	29 (5.4%)		96 (8.4%)	88 (7.4%)
pT4	1 (0%)	0 (0%)		1 (0.1%)	0 (0%)
N stage (any neoadjuvant)			0.681		
cN0	190 (32.9%)	67 (34.5%)		83 (31.1%)	107 (34.5%)
cN1 + cN2 + cN3	387 (67.1%)	127 (65.5%)		184 (68.9%)	203 (65.5%)
N stage (no neoadjuvant)			0.327		
pN0 + pN0(i+)	1111 (47.5%)	244 (45.2%)		532 (46.5%)	579 (48.5%)
pN1 + pN1mi + pN2 + pN3	1227 (52.5%)	296 (54.8%)		613 (53.5%)	614 (51.5%)
Hormone receptor status			0.004		
ER-negative and PgR-negative	859 (29.5%)	257 (35%)		415 (29.4%)	444 (29.5%)
ER-positive and/or PgR-positive	2056 (70.5%)	477 (65%)		997 (70.6%)	1059 (70.5%)
HER2 status			0.765		
Negative	2417 (82.9%)	612 (83.4%)		1169 (82.8%)	1248 (83%)
Positive	498 (17.1%)	122 (16.6%)		243 (17.2%)	255 (17%)
Most extensive primary surgery			0.473		
Mastectomy, NOS	1458 (50%)	378 (51.5%)		734 (52%)	724 (48.2%)
Partial mastectomy/lumpectomy/excisional biopsy	1457 (50%)	356 (48.5%)		678 (48%)	779 (51.8%)
Perioperative chemotherapy			<0.001		
Missing	0 (0%)	1 (0.1%)		0 (0%)	0 (0%)
No	310 (10.6%)	81 (11%)		142 (10.1%)	168 (11.2%)
Yes—Any neoadjuvant	575 (19.7%)	193 (26.3%)		267 (18.9%)	308 (20.5%)
Yes—Postoperative only	2030 (69.6%)	459 (62.5%)		1003 (71%)	1027 (68.3%)
Perioperative hormone therapy			0.003		
No	1087 (37.3%)	322 (43.9%)		527 (37.3%)	560 (37.2%)
Yes—Any neoadjuvant	7 (0.2%)	1 (0.1%)		3 (0.2%)	4 (0.3%)
Yes—Postoperative only	1821 (62.5%)	411 (56%)		882 (62.5%)	939 (62.4%)
Perioperative Trastuzumab			0.799		
No	2411 (82.7%)	610 (83.1%)		1167 (82.6%)	1244 (82.8%)
Yes	504 (17.3%)	124 (16.9%)		245 (17.4%)	259 (17.2%)
Sample for rs11212617 SNP			<0.001		
Available	2747 (94.2%)	579 (78.9%)		1329 (94.1%)	1418 (94.3%)
Unavailable	168 (5.8%)	155 (21.1%)		83 (5.9%)	85 (5.7%)
rs11212617 SNP			0.396		
AA	823 (30%)	179 (30.9%)		401 (30.2%)	422 (29.8%)
CA	1347 (49%)	267 (46.1%)		657 (49.4%)	690 (48.7%)
CC	577 (21%)	133 (23%)		271 (20.4%)	306 (21.6%)

Mean age of included subjects was 52 years, the majority (91%) were white with clinical or pathologic T2 or T3 tumors (67.4% metformin, 65.3% placebo) that were node-positive (56.4% metformin, 54.4% placebo), hormone receptor-positive (70.6% metformin, 70.5% placebo), and HER2 negative (82.8% metformin, 83% placebo). The majority received adjuvant chemotherapy (89.9% metformin, 88.8% placebo) and hormone therapy (62.7% in both arms). Adjuvant trastuzumab use was similar in both arms (17.4% metformin, 17.2% placebo). Just over one-third (34.3% metformin, 33.8% placebo) had BMI ≥ 30 kg/m^2^ at baseline.

The AA genotype of the rs11212617 SNP was present in 30% of the metabolic population, AC in 49% and CC in 21%; by-arm distributions were similar. A and C allelic frequencies were 0.54 and 0.46, respectively, in accordance with Hardy Weinberg equilibrium (*X*^2^ = 0.35, *p* = 0.55). When patients were classified by self-reported race/ethnicity, the genotype frequencies differed significantly among groups (*p* ≤ 0.001), A being present in the majority of Whites (80.1%) and C in the majority of African Americans (86.3%). Specific frequencies for AA, AC, and CC were 30.7%, 49.4%, and 19.9% in 2530 White, 25.4%, 50.7%, and 23.9% in 67 Asian, 13.7%, 36.8%, and 49.5% in 95 African American, 20.8%, 62.5%, and 16.7% in 24 American Indian, Alaskan Native, Native Hawaiian or other Pacific Islander subjects, and 38.7%, 41.9%, and 19.4% in 31 subjects with non-reported ethnicity, respectively.

### Baseline and 6-month change in metabolic factors

Baseline values, raw differences and relative change from baseline to 6 months are shown in Table 2 for body size and circulating metabolic factors. Month 6 standardized metformin to placebo ratios, adjusted for baseline differences in the variable, BMI and age are also shown. Baseline values of all variables were similar in metformin vs placebo arms. At 6 months, there were significant raw and relative improvements in all variables in metformin versus placebo subjects. Metformin subjects lost a median of 1.4 kg (vs 0.5 kg gain in placebo subjects, *p* < 0.0001; relative change −2% metformin vs +1% placebo, *p* < 0.0001). In univariable analyses, metformin (vs placebo) subjects experienced reductions in glucose (raw change −0.1 vs +0.1 mmol/L, *p* < 0.0001; relative change −2% vs +1%, *p* < 0.0001), insulin (raw change −7 vs +2 pmol/L, *p* < 0.0001; relative change −11% vs +6%, *p* < 0.0001), HOMA (raw change −0.2 vs +0.1, *p* < 0.0001; relative change −10% vs +11%, *p* < 0.0001), leptin (raw change −0.8 vs +1.1 ng/ml, *p* < 0.0001; relative change −10% vs +12%, *p* < 0.0001), and hsCRP (raw change −0.1 vs +0.1 μg/L, *p* < 0.0001; relative change −9% vs +10%, *p* < 0.0001). After adjustment for baseline variable levels, BMI and age, the standardized 6-month metformin to placebo ratios were 0.97 for weight or BMI, 0.98 for glucose, 0.85 for insulin, 0.83 for HOMA, 0.80 for leptin, and 0.84 for hsCRP (all *p* < 0.0001 compared to a ratio of 1). This reflects, for example, 6-month insulin levels being 15% lower in the metformin arm than in the placebo arm when standardized to patients with the same baseline insulin, age, and BMI.

**Table 2:** Metabolic factors at baseline and change after 6 months of treatment with the study drug (metformin or placebo).

		Baseline measurements		Change from baseline (B) to month 6 (F)				
Metabolic factor	Measure	Metformin	Placebo	Dimension	Metformin (M)	Placebo (P)		*P*
Weight (kg)	# Patients	1412	1503		1394	1499		
	Mean (SD)	76.8 (17.7)	75.4 (16.8)	Raw difference F−B	−1.6 (3.8)	0.6 (4.7)		
	Median (Q1–Q3)	73.6 (64.4–86.0)	72.5 (63.1–84.5)		−1.4 (−3.6–0.5)	0.5 (−1.3–2.5)		<0.0001
	Ratio			Relative change^a^	−2%	+1%		<0.0001
	Ratio (95% CI)			Standardized ratio M/P^b^	0.97 (0.97–0.98)			<0.0001
BMI (kg/m^2^)	# Patients	1412	1503		1394		1499	
	Mean (SD)	28.7 (6.6)	28.4 (6.1)	Raw difference F−B	−0.6 (1.4)		0.2 (1.7)	
	Median (Q1–Q3)	27.4 (24.0–31.9)	27.3 (24.1–31.7)		−0.5 (−1.4–0.2)		0.2 (−0.5–1.0)	<0.0001
	Ratio			Relative change^a^	−2%		+1%	<0.0001
	Ratio (95% CI)			Standardized ratio M/P^b^	0.97 (0.97–0.98)			<0.0001
Glucose (mmol/L)	# Patients	1412	1503		1345		1442	
	Mean (SD)	5.2 (0.6)	5.2 (0.6)	Raw difference F−B	−0.1 (0.6)		0 (0.6)	
	Median (Q1–Q3)	5.2 (4.8–5.6)	5.2 (4.8–5.5)		−0.1 (−0.4–0.2)		0.1 (−0.3–0.3)	<0.0001
	Ratio			Relative change^a^	−2%		+1%	<0.0001
	Ratio (95% CI)			Standardized ratio M/P^b^	0.98 (0.97–0.99)			<0.0001
Insulin (pmol/L)	# Patients	1405	1498		1395		1491	
	Mean (SD)	83.0 (85.1)	80.2 (85.4)	Raw difference F−B	−8.6 (88.2)		5.6 (89)	
	Median (Q1–Q3)	60 (41–95)	59 (40–94)		−7 (−25–11)		2 (−14–21)	<0.0001
	Ratio			Relative change^a^	−11%		+6%	<0.0001
	Ratio (95% CI)			Standardized ratio M/P^b^	0.85 (0.82–0.88)			<0.0001
HOMA	# Patients	1041	1108		899		964	
	Mean (SD)	2.6 (2.2)	2.5 (2.8)	Raw difference F−B	−0.2 (2.5)		0.4 (2.9)	
	Median (Q1–Q3)	1.9 (1.3–3.1)	1.9 (1.2–2.9)		−0.2 (−0.8–0.4)		0.1 (−0.4–0.8)	<0.0001
	Ratio			Relative change^a^	−10%		+11%	<0.0001
	Ratio (95% CI)			Standardized ratio M/P^b^	0.83 (0.79–0.87)			<0.0001
Leptin (ng/ml)	# Patients	1412	1501		1412		1500	
	Mean (SD)	16.8 (16.6)	16.4 (16)	Raw difference F−B	−1 (11.3)		1.9 (11.2)	
	Median (Q1–Q3)	12.7 (6.5–21.9)	12.4 (6.1–21.7)		−0.8 (−4.6–2.1)		1.1 (−1.7–5.2)	<0.0001
	Ratio			Relative change^a^	−10%		+12%	<0.0001
	Ratio (95% CI)			Standardized ratio M/P^b^	0.80 (0.77–0.83)			<0.0001
hsCRP (μg/L)	# Patients	1412	1501		1411		1501	
	Mean (SD)	3.0 (6.2)	2.7 (4.3)	Raw difference F−B	0.3 (10.8)		0.5 (6.8)	
	Median (Q1–Q3)	1.3 (0.5–3.2)	1.3 (0.5–3.1)		−0.1 (−0.7–0.4)		0.1 (−0.4–0.7)	<0.0001
	Ratio			Relative change^a^	−9%		+10%	<0.0001
	Ratio (95% CI)			Standardized ratio M/P^b^	0.84 (0.78–0.80)			<0.0001

*B* baseline, *F* follow-up, *M* metformin, *P* placebo, *SD* standard deviation, *Q1* 25th percentile, *Q3* 75th percentile, *CI* confidence interval.

^a^Relative change: raw change expressed as a percentage of baseline, i.e., (F − B)/B × 100.

^b^Standardized ratio M/P: the relative levels in the two arms at 6 months standardized to remove the baseline differences in the variable, age and BMI, obtained from an adjusted regression model for log-change in the metabolic variable.

**Fig. 2:**
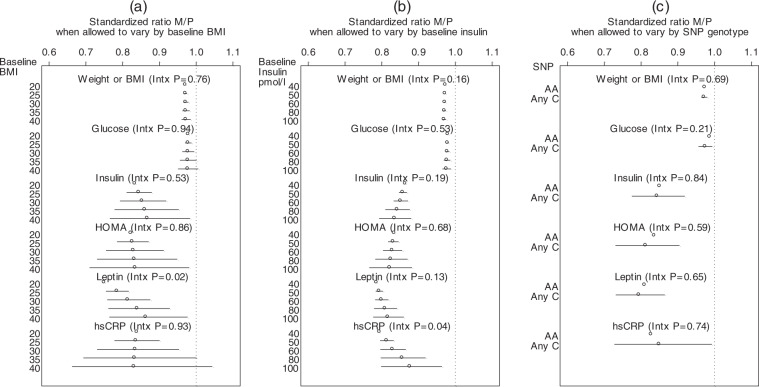
Assessment of differential study drug effects on the metabolic outcomes weight/BMI, glucose, insulin, HOMA, leptin, and hsCRP, by baseline BMI, insulin, and the rs11212617 SNP. Depicted is the standardized metformin to placebo ratio of the metabolic factor at 6 months, with 95% confidence intervals, obtained from adjusted interaction regression models. BMI body mass index, SNP single-nucleotide polymorphism, Intx interaction. **a** Interaction of study drug by baseline BMI (continuous). **b** Interaction of study drug by baseline insulin (continuous). **c** Interaction of study drug by genotype any C versus AA of the rs11212617 SNP.

There was little evidence of a significant interaction of change in these variables with baseline BMI or insulin. The standardized metformin to placebo ratios at 6 months for each variable according to baseline BMI and baseline insulin are shown in Fig. 2 panels a and b. As can be seen, potential quantitative interactions were identified for baseline BMI with leptin (interaction *p* = 0.02, standardized metformin to placebo ratio 0.75 when BMI = 20 kg/m^2^ and 0.81 for BMI = 30 kg/m^2^) and for baseline insulin with hsCRP (interaction *p* = 0.04, standardized ratio 0.79 for insulin = 40 pmol/L and 0.88 for insulin = 100 pmol/L). Similarly, there was no evidence that change in body size or blood variables differed in those receiving adjuvant endocrine therapy vs no (all interaction *P* ≥ 0.22, see Supplemental Table 1).

### rs11212617 SNP

At baseline, a pattern of increasing weight, BMI, and leptin was seen according to the number of rs11212617 C alleles (Table 3): median weights for the three groups AA, AC, and CC are 72.0, 73.3, and 75.4 kg (*p* = 0.01); median BMI 27.0, 27.4, and 28.2 kg/m^2^ (*p* = 0.02); median leptin 12.0, 12.6, and 13.4 ng/ml (*p* = 0.05).

**Table 3:** Metabolic factors at baseline broken down by the rs11212617 SNP.

Metabolic factor	Measure	AA	AC	CC	*P*^a^	AA	Any C	*P*^a^
Weight	# Patients	823	1347	577		823	1924	
(kg)	Mean (SD)	75.4 (17.1)	76.1 (16.8)	78.1 (18.7)		75.4 (17.1)	76.7 (17.4)	
	Median (Q1–Q3)	72.0 (63.4–83.9)	73.3 (64–85.5)	75.4 (65–87.8)	0.01	72.0 (63.4–83.9)	73.9 (64.2–86)	0.03
BMI	# Patients	823	1347	577		823	1924	
(kg/m^2^)	Mean (SD)	28.3 (6.3)	28.5 (6.1)	29.3 (7)		28.3 (6.3)	28.8 (6.4)	
	Median (Q1–Q3)	27.0 (23.8–31.6)	27.4 (24.2–31.5)	28.2 (24.3–32.9)	0.02	27.0 (23.8–31.6)	27.6 (24.2–32)	0.06
Glucose	# Patients	823	1347	577		823	1924	
(mmol/L)	Mean (SD)	5.2 (0.6)	5.2 (0.5)	5.2 (0.6)		5.2 (0.6)	5.2 (0.6)	
	Median (Q1–Q3)	5.2 (4.8–5.6)	5.2 (4.8–5.5)	5.2 (4.8–5.6)	0.40	5.2 (4.8–5.6)	5.2 (4.8–5.5)	0.22
Insulin	# Patients	820	1344	573		820	1917	
(pmol/L)	Mean (SD)	76.3 (69.1)	83.0 (91.2)	86.2 (93.3)		76.3 (69.1)	83.9 (91.8)	
	Median (Q1–Q3)	59 (40–94)	60 (41–94)	60 (40–97)	0.76	59 (40–94)	60 (40–94)	0.47
HOMA	# Patients	628	987	416		628	1403	
	Mean (SD)	2.5 (1.9)	2.6 (2.9)	2.6 (2.5)		2.5 (1.9)	2.6 (2.8)	
	Median (Q1–Q3)	2.0 (1.3–3.1)	1.9 (1.2–3)	1.8 (1.2–3)	0.74	2.0 (1.3–3.1)	1.9 (1.2–3)	0.50
Leptin	# Patients	823	1346	576		823	1922	
(ng/ml)	Mean (SD)	15.9 (16.5)	16.2 (14.5)	18.7 (19.6)		15.9 (16.5)	17 (16.2)	
	Median (Q1–Q3)	12.0 (6–21.3)	12.6 (6.4–21.2)	13.4 (6.2–24.4)	0.05	12.0 (6–21.3)	12.7 (6.3–22.1)	0.07
hsCRP	# Patients	823	1346	576		823	1922	
(μg/L)	Mean (SD)	3.0 (7.2)	2.9 (4.7)	2.8 (3.9)		3.0 (7.2)	2.9 (4.5)	
	Median (Q1–Q3)	1.2 (0.5–3)	1.3 (0.5–3.3)	1.5 (0.6–3.3)	0.25	1.2 (0.5–3)	1.4 (0.5–3.3)	0.28

Three SNP categories are shown on the left and two on the right.

*SD* standard deviation, *Q1* 25th percentile, *Q3* 75th percentile.

^a^The null hypothesis of no difference was tested using Kruskal–Wallis tests (3 groups) and Wilcoxon rank-sum tests (2 groups).

Examination of interactions between the study drug (metformin versus placebo) and the SNP status (any C versus AA) showed no evidence that metformin was more effective on body size or metabolic factors when the C allele was present (Fig. 2c, all interaction *P* > 0.21).

## Discussion

Metformin (versus placebo) was associated with improvement in body size as well as a range of circulating metabolic markers in the non-diabetic population that was enrolled onto MA.32. For the most part, these improvements with metformin were independent of baseline BMI and insulin levels and did not vary by rs11212617 genotype. Although the borderline significant interactions of baseline BMI with change in leptin and of insulin with change in hsCRP may have been due to chance (multiple interactions were tested for multiple variables) they warrant evaluation in future studies; it is important to note that these potential interactions were quantitative, not qualitative with metformin (vs placebo) benefits being consistently observed. These findings suggest that the improvement in metabolic factors seen with metformin is broad-based. Furthermore, the relative changes in metformin vs placebo subjects that we identified (e.g., a 15% lower insulin in metformin relative to placebo subjects at 6 months) were clinically significant and consistent with our hypothesized effects. Together, our findings support the study of metformin in both metabolically healthy and unhealthy BC patients and they are consistent with potential beneficial indirect effects of metformin on BC outcomes. In our upcoming efficacy analysis of MA.32 we will analyze the extent to which metabolic response to metformin is associated with BC outcomes. Our observations also suggest metformin may exert beneficial effects on cardiovascular and metabolic outcomes; this will be examined in our upcoming analysis.

Our results are consistent with two prior reports, in which metformin 500 mg po tid was given for 6 months to BC survivors^17^ or for 2–3 weeks in a BC neoadjuvant window of opportunity study^6^, with change measurements taken while subjects were still taking metformin. In both studies, significant improvements were identified in weight, BMI and the metabolic factors reported here. In the neoadjuvant study, reductions in intra-tumoral ki67 that were independent of baseline BMI and HOMA were also seen. Similar improvements in metabolic status were reported by Kalinsky et al.^18^ in another neoadjuvant window of opportunity study in which subjects received metformin 500 mg in the morning and 1000 mg in the evening for a median of 23 days, although no associated reductions were seen in intra-tumoral ki67. In contrast, in a third BC neoadjuvant window of opportunity study^19^, there were no improvements in metabolic variables in patients receiving metformin 1700 mg once daily after dinner for 4 weeks when metformin was discontinued 36–48 h before final measurements. Given a metformin half-life of ~6 h, the once-daily dosing schedule, coupled with the longer interval between drug discontinuation and final measurements may have obscured metabolic effects. Regardless, significant reductions in intra-tumoral ki67 were seen in individuals receiving metformin who had baseline insulin resistance (as defined by HOMA).

Our failure to identify an association of metabolic improvement with the rs11212617 SNP genotype was somewhat unexpected since some prior reports^11,12,14^ have identified the C allele to be associated with enhanced glycemic response to metformin and/or higher plasma levels of the drug in diabetic patients. It is possible the differential glycemic effect of the C vs A allele is present only in diabetic subjects (MA.32 subjects were non-diabetic). The C allele has been associated with enhanced pathologic complete response to neoadjuvant chemotherapy/HER2 targeted treatment in non-diabetic HER2 + BC subjects^15^ receiving metformin; this SNP associated effect may have been mediated by mechanisms that differed from the metabolic mechanisms we investigated here. Our observation that the C allele is the major allele in African Americans (versus A in White/Caucasian populations) is novel. As a result, it is possible metformin effects or metformin toxicity will differ by race/ethnicity. Indeed, increased treatment success (reductions in HbA1C) has been reported in African Americans with type 2 diabetes receiving metformin^20^; African Americans in the Diabetes Prevention Program randomized to metformin exhibited greater reductions (though non-significant) in diabetes incidence when compared to White subjects^21^. We plan to examine whether rs11212617 status and race are predictors of metformin effect on BC outcomes in MA.32 and we will examine metformin toxicity in relation to SNP status.

Strengths of our study include the prospective randomized design, large sample size, and standardization of blood handling and analysis. Limitations include the lack of data on more sensitive measures of insulin sensitivity, such as the frequently sampled intravenous glucose tolerance test. Furthermore, although subjects were required to be receiving study drug at the time of the 6-month measurement, some subjects may not have fully complied with drug dosing in the days leading up to the 6-month blood draw. We were not able to explore the potential contribution of metformin-associated toxicity to the changes in body size and metabolic factors we have studied; data on toxicity and compliance will be available after the planned efficacy analysis and these issues will be explored at that time. In addition, because information on diet and physical activity was not available on the majority of subjects (>75%), we were not able to explore the impact of lifestyle on change in body size and metabolic factors.

In conclusion, the administration of metformin 850 mg po bid to non-diabetic early-stage breast cancer patients led to weight loss and improved metabolic health. These improvements were largely independent of baseline BMI and insulin and did not differ by rs11212617 SNP status. They provide support for a potential indirect of metformin on breast cancer outcomes (mediated by reductions in insulin and other metabolic markers). Their potential contribution to metformin effects on breast cancer outcomes, as well as on cardiovascular disease and diabetes, will be examined in upcoming efficacy analyses, strengthening the clinical utility of our results.

## Methods

### Study design

The CCTG MA.32 Clinical Trial (Clinical Trials.gov identifier: NCT01101438; http://clinicaltrials.gov/show/NCT01101438, first posted April 12, 2010) is a Phase III, randomized trial that enrolled 3649 non-diabetic subjects between 2010 and 2013; subjects received standard surgical, chemotherapeutic (completed at least one month prior to enrollment), hormonal, biologic, and radiation therapy for a T1-3, N0-3, M0 BC diagnosed during the previous year. Subjects with T1c N0 BC were eligible if they had at least one of histologic grade III, lymphovascular invasion, negative estrogen (ER) and progesterone (PgR) receptors, HER2 positivity, Oncotype Recurrence Score ≥25 or Ki-67 over 14%. In May 2012, after 2382 subjects were enrolled, eligibility criteria were amended to mandate triple negative (ER-negative, PgR-negative, HER2 negative) status for patients with T1cN0 disease and at least one of the above adverse tumor characteristic for patients with T2N0 tumors. Participants were required to have a fasting glucose of 7.0 mmol/L or lower; those with a history of diabetes, lactic acidosis, current use of diabetes medication, breast cancer recurrence or previous invasive cancer, excessive alcohol intake, or marked hepatic, kidney, or cardiac dysfunction were excluded.

Eligible subjects were randomly assigned (1:1, using computer-generated randomization) in a double-blind fashion to metformin 850 mg caplet po bid or an identical placebo po bid for 5 years, including a 4-week ramp-up of one caplet per day. Height and weight were measured (in indoor clothing, without shoes) at study centers at baseline and weight at 6 months. The primary study outcome, invasive disease-free survival, as well as secondary outcomes, including overall survival and breast cancer-free interval, have not yet been reported.

The study protocol was approved by institutional review boards of participating institutions, including the NCI (US) Central Institutional Review Board and Mount Sinai Hospital (Ontario Cancer Research Ethics Board). All patients provided written informed consent to participate.

### Laboratory analyses

At baseline (before starting study therapy) blood was drawn into a lithium heparin tube after an overnight fast of at least 12 h and centrifuged at 1500 rpm for 10 min; plasma was aliquoted and frozen within 30 min of collection at −80 °C at the local center. Aliquots were transported on dry ice to the central repository at CCTG in Kingston, Canada. Specimens were re-transported on dry ice to Mount Sinai Hospital in Toronto for analysis. Paired bloods (baseline, 6 months) were assayed (blinded to treatment allocation, without dilution) in batches with 10% random repeats for insulin (Roche ElectroChemiLuminescence Immunoassay (ECLIA), leptin (Luminex Milliplex MAP assay), and hsCRP (Roche, particle-based immunoturbidimetric assay). Blood was analyzed in 2014–2015. Intra-assay coefficients of variability were 3%, 3%, and 4% for insulin, leptin, and hsCRP, respectively. Glucose was analyzed at local clinical laboratories immediately after collection. HOMA (a marker of insulin resistance) was calculated from glucose and insulin levels when both were measured on the same day [glucose (mg/dl) × insulin (pmol/L)/22.5]^16^.

Blood for genomic analysis was drawn into EDTA tubes that were aliquoted into 1.5 ml cryovials and stored as described above. One aliquot was sent on dry ice for genomic DNA extraction and genotyping for the SNP rs11212617 (Chr11(GRCh38):g.108412434C>A) at The Centre for Applied Genomics, Hospital for Sick Children, Toronto, Canada, using a QIAsymphony magnetic bead DNA extractor (Qiagen, Germany) and PCR primers (5′ACAAACAGGAAACAATTACAAATACAATAAAT3′ and 5′TTAAAGTGGGTTGCTTGTGGATAA3′) with TaqMan® 100 mM dual-label MGB probes AGATCAGAGA**C**TGTCAGAGC and AGATCAGAGA**A**TGTCAGAGC (Applied Biosystems™, ThermoFisher Scientific, Waltham, MA, USA). The reaction mix consisted of 5 μl TaqMan® Genotyping Master Mix (Life Technologies), 1 μl of each PCR primer (each at 10 mM), 0.02 μl of each probe, 2.0 μl water, and 20–50 ng of DNA template. Samples were analyzed using the ViiA™ 7 Real-Time PCR System (Applied Biosystems™) and analyzed using ViiA™ 7 software. Each genotyping run contained 92 study samples along with Coriell reference samples NA12878 (C/A), NA12813 (A/A), NA19240 (C/C), and a no template control.

### Statistical analyses

Statistical analyses were conducted by Drs. Bingshu Chen (CCTG) and Marguerite Ennis using SAS version 9.2. Patient and tumor characteristics at baseline were tabulated by study arm and compared using *χ*^2^ tests for categorical variables and Wilcoxon rank-sum tests for continuous variables. These tests were performed because selection criteria for this study included post-randomization criteria.

Change from baseline (B) to follow-up (F) after 6 months of treatment with metformin or placebo was assessed. Summary statistics for raw change (follow-up minus baseline, F − B) were tabulated by arm and compared using Wilcoxon rank-sum tests. All the metabolic factors had skew distributions which were greatly improved by applying log transformations. The average log-change (average of log(F) − log(B)) was calculated and the arms compared via *t*-tests. An effect size measure was obtained by back-transforming the log-change averages to geometric means F/B and calculating percent relative change as (F − B)/B × 100. Thus the relative change denotes raw within-arm change. Further comparisons that adjusted for baseline age, BMI, and baseline levels of the metabolic variable were performed using linear regression models with log-change as an outcome. Results are presented as standardized ratios by back-transforming the study arm parameter. Because of the baseline adjustment, these ratios give the relative levels in the two arms at 6 months “standardized” to remove any baseline differences between the two arms in the variable, age and BMI. Note that when using ratios the same results are obtained for BMI as for weight because height cancels out in the BMI ratios.

The above regression models were expanded with suitable interaction terms to explore whether the study drug had a differential effect on a metabolic outcome depending on (1) baseline BMI level, (2) baseline insulin level, and (3) the genotype any C versus AA of the rs11212617 SNP.

Measures of effect size and uncertainty were provided. A *p* value ≤ 0.05 was considered statistically significant and all tests were two-sided.

### Reporting summary

Further information on research design is available in the Nature Research Reporting Summary linked to this article.

## Supplementary information

Supplementary Information

Reporting Summary

## Data Availability

The data generated and analyzed during this study are described in the following data record: 10.6084/m9.figshare.14447598^22^. The SNP data are openly available as part of the data record. The primary efficacy analysis will be available from the Canadian Cancer Trials Group (Kingston, Ontario) after the results of the analysis have been published. These data will be uploaded to the NCI data archive website: https://nctn-data-archive.nci.nih.gov/view-trials and will be searchable via NCT trial number NCT01101438. As of April 2021, the group is working towards this publication. Further details can be requested from the corresponding author. The clinical data are not publicly available for the following reason: data contain information that could compromise research participant privacy.
