# Supplementary material for: Effect of metformin versus placebo on metabolic factors in the MA.32 randomized breast cancer trial
Source: NPJ Breast Cancer. 2021 Jun 8;7:74. doi: 10.1038/s41523-021-00275-z (PMC8187713; doi:10.1038/s41523-021-00275-z)
Supplement: Supplementary file 1 — Supplementary Information [file 41523_2021_275_MOESM1_ESM.pdf]

Supplemental Table 1. Assessment of differential effects of study drug by perioperative hormone therapy on the metabolic outcomes weight/BMI, glucose, insulin, HOMA, leptin and hsCRP. M/P is the standardized metformin to placebo ratio of the metabolic factor at 6 months obtained from adjusted interaction regression models, with 95% confidence intervals.

	Standardized ratio M/P		Interaction assessment	
Metabolic factor	Therapy not performed	Therapy performed	Ratio of two M/P ratios	Interaction P (2-sided)
Weight or BMI	0.97	0.97 (0.96 - 0.98)	1.00 (0.99 - 1.01)	0.86
Glucose	0.99	0.97 (0.96 - 0.99)	0.99 (0.97 - 1.01)	0.22
Insulin	0.87	0.84 (0.78 - 0.91)	0.97 (0.90 - 1.05)	0.46
HOMA	0.85	0.81 (0.74 - 0.90)	0.95 (0.86 - 1.05)	0.34
Leptin	0.80	0.80 (0.74 - 0.87)	1.00 (0.92 - 1.08)	0.96
hsCRP	0.87	0.81 (0.71 - 0.94)	0.93 (0.81 - 1.07)	0.31

BMI = Body Mass Index
