# Supplementary material for: Effect of metformin versus placebo on metabolic factors in the MA.32 randomized breast cancer trial
Source: NPJ Breast Cancer. 2021 Jun 8;7:74. doi: 10.1038/s41523-021-00275-z (PMC8187713; doi:10.1038/s41523-021-00275-z)
Supplement: Supplementary file 2 — Reporting Summary [file 41523_2021_275_MOESM2_ESM.pdf]

nature research

Corresponding author(s): Pamela Goodwin

Last updated by author(s): Apr 22, 2021

Reporting Summary

Nature Research wishes to improve the reproducibility of the work that we publish. This form provides structure for consistency and transparency in reporting. For further information on Nature Research policies, see our Editorial Policies and the Editorial Policy Checklist.

Statistics

For all statistical analyses, confirm that the following items are present in the figure legend, table legend, main text, or Methods section.

n/a	Confirmed	
☐	☒	The exact sample size (*n*) for each experimental group/condition, given as a discrete number and unit of measurement
☐	☒	A statement on whether measurements were taken from distinct samples or whether the same sample was measured repeatedly
☐	☒	The statistical test(s) used AND whether they are one- or two-sided<br>*Only common tests should be described solely by name; describe more complex techniques in the Methods section.*
☐	☒	A description of all covariates tested
☐	☒	A description of any assumptions or corrections, such as tests of normality and adjustment for multiple comparisons
☐	☒	A full description of the statistical parameters including central tendency (e.g. means) or other basic estimates (e.g. regression coefficient) AND variation (e.g. standard deviation) or associated estimates of uncertainty (e.g. confidence intervals)
☐	☒	For null hypothesis testing, the test statistic (e.g. *F*, *t*, *r*) with confidence intervals, effect sizes, degrees of freedom and *P* value noted<br>*Give P values as exact values whenever suitable.*
☒	☐	For Bayesian analysis, information on the choice of priors and Markov chain Monte Carlo settings
☒	☐	For hierarchical and complex designs, identification of the appropriate level for tests and full reporting of outcomes
☐	☒	Estimates of effect sizes (e.g. Cohen's *d*, Pearson's *r*), indicating how they were calculated

Our web collection on statistics for biologists contains articles on many of the points above.

Software and code

Policy information about availability of computer code

Data collection	snp results will be made available without associated clinical data
Data analysis	SAS version 9.2 was used

For manuscripts utilizing custom algorithms or software that are central to the research but not yet described in published literature, software must be made available to editors and reviewers. We strongly encourage code deposition in a community repository (e.g. GitHub). See the Nature Research guidelines for submitting code & software for further information.

Data

Policy information about availability of data

All manuscripts must include a data availability statement. This statement should provide the following information, where applicable:

- Accession codes, unique identifiers, or web links for publicly available datasets
- A list of figures that have associated raw data
- A description of any restrictions on data availability

The datasets generated during and/or analysed during the current study will be available from the Canadian Cancer Trials Group (Kingston, Ontario) after the results of the primary efficacy analysis have been published. Snp results will be made available.

April 2020

Field-specific reporting

Please select the one below that is the best fit for your research. If you are not sure, read the appropriate sections before making your selection.

☒ Life sciences ☐ Behavioural & social sciences ☐ Ecological, evolutionary & environmental sciences

For a reference copy of the document with all sections, see nature.com/documents/nr-reporting-summary-flat.pdf

Life sciences study design

All studies must disclose on these points even when the disclosure is negative.

Sample size	This substudy used all available subjects with fasting blood at baseline and 6 months (2915 of 3649 subjects in the parent trial).
Data exclusions	No data were excluded
Replication	No replications were undertaken
Randomization	Computer generated randomization stratified for : (i) BMI </> 30 kg/m2, (ii) ER/PgR + vs -, (iii) Her2 + vs -, (iv) chemotherapy (any vs none)
Blinding	Investigators, laboratories and subjects were blinded to treatment allocation.

Reporting for specific materials, systems and methods

We require information from authors about some types of materials, experimental systems and methods used in many studies. Here, indicate whether each material, system or method listed is relevant to your study. If you are not sure if a list item applies to your research, read the appropriate section before selecting a response.

Materials & experimental systems

n/a	Involved in the study	
☒	☐	Antibodies
☒	☐	Eukaryotic cell lines
☒	☐	Palaeontology and archaeology
☒	☐	Animals and other organisms
☐	☒	Human research participants
☐	☒	Clinical data
☒	☐	Dual use research of concern

Methods

n/a	Involved in the study	
☒	☐	ChIP-seq
☒	☐	Flow cytometry
☒	☐	MRI-based neuroimaging

Human research participants

Policy information about studies involving human research participants

Population characteristics

Mean age of included subjects was 52 years, the majority (91%) were white with clinical or pathologic T2 or T3 tumors (67.4% metformin, 65.3% placebo) that were node positive (56.4% metformin, 54.4% placebo), hormone receptor positive (70.6% metformin, 70.5% placebo) and HER2 negative (82.8% metformin, 83% placebo). The majority received adjuvant chemotherapy (89.9% metformin, 88.8% placebo) and hormone therapy (62.7% in both arms). Adjuvant trastuzumab use was similar in both arms (17.4% metformin, 17.2% placebo). Just over one-third (34.3% metformin, 33.8% placebo) had BMI > 30 kg/m2 at baseline.
The AA genotype of the rs11212617 SNP was present in 30% of the metabolic population, AC in 49% and CC in 21%; by-arm distributions were similar. A and C allelic frequencies were 0.54 and 0.46, respectively, in accordance with Hardy Weinberg equilibrium (X2=0.35, p=0.55). When patients were classified by self-reported race/ethnicity, the genotype frequencies differed significantly among groups (p=<0.001), A being present in the majority of Whites (80.1%) and C in the majority of African Americans (86.3%). Specific frequencies for AA, AC, and CC were 30.7%, 49.4%, and 19.9% in 2530 White, 25.4%, 50.7%, and 23.9% in 67 Asian, 13.7%, 36.8%, and 49.5% in 95 African American, 20.8%, 62.5%, and 16.7% in 24 American Indian, Alaskan Native, Native Hawaiian or other Pacific Islander subjects, and 38.7%, 41.9%, and 19.4% in 31 subjects with non-reported ethnicity, respectively.

Table 1. Baseline patient and tumour characteristics
Included and Excluded from Metabolic Population Metabolic Population
Study Arms
Included
N=2915 Excluded
N=734 P Metf
N=1412 Plac
N=1503

Treatment arm <0.001
Metformin 1412 (48.4%) 412 (56.1%)
Placebo 1503 (51.6%) 322 (43.9%)

AGE (years) 0.86
Mean 52.39 52.3 52.04 52.72
SD 9.999 10.45 9.981 10.01
BMI kg/m2 0.35
< 25 952 (32.7%) 222 (30.2%) 460 (32.6%) 492 (32.7%)
>= 25 and <30 963 (33.0%) 242 (33%) 460(32.6%) 503 (33.5%)
>= 30 1000 (34.3%) 270 (36.8%) 492 (34.8%) 508 (33.8%)

RACE <0.001
Asian 78 (2.7%) 21 (2.9%) 35 (2.5%) 43 (2.9%)
Black or African American 112 (3.8%) 55 (7.5%) 54 (3.8%) 58 (3.9%)
America Indian or Alaska Native or Native Hawaiian or Pacific Islander 26 (0.9%) 4 (0.5%) 13 (0.9%) 13 (0.9%)
White 2667 (91.5%) 637 (86.8%) 1295 (91.7%) 1372 (91.3%)
Not reported (or refused) or Unknown 32 (1.1%) 17 (2.3%) 15 (1.1%) 17 (1.1%)

T STAGE (any neoadjuvant) 0.294
cT1a+cT1b+cT1c 57 (9.9%) 27 (13.9%) 18 (6.7%) 39 (12.6%)
cT2 329 (57%) 105 (54.1%) 162 (60.7%) 167 (53.9%)
cT3 191 (33.1%) 62 (32%) 87 (32.6%) 104 (33.5%)

T STAGE (no neoadjuvant) 0.187
pT1a+pT1b+pT1c+pT1mic 926 (39.6%) 229 (42.4%) 443 (38.7%) 483 (40.5%)
pT2 1227 (52.5%) 282 (52.2%) 605 (52.8%) 622 (52.1%)
pT3 184 (7.9%) 29 (5.4%) 96 (8.4%) 88 (7.4%)
pT4 1 (0%) 0 (0%) 1 (0.1%) 0 (0%)

N STAGE (any neodadjuvant) 0.681
cN0 190 (32.9%) 67 (34.5%) 83 (31.1%) 107 (34.5%)
cN1+cN2+cN3 387 (67.1%) 127 (65.5%) 184 (68.9%) 203 (65.5%)

N STAGE (no neoadjuvant) 0.327
pN0+pN0(i+) 1111 (47.5%) 244 (45.2%) 532 (46.5%) 579 (48.5%)
pN1+pN1mi+pN2+pN3 1227 (52.5%) 296 (54.8%) 613 (53.5%) 614 (51.5%)

HORMONE RECEPTOR STATUS 0.004
ER-negative and PgR-negative 859 (29.5%) 257 (35%) 415 (29.4%) 444 (29.5%)
ER-positive and/or PgR-positive 2056 (70.5%) 477 (65%) 997 (70.6%) 1059 (70.5%)

HER2 0.765
Negative 2417 (82.9%) 612 (83.4%) 1169 (82.8%) 1248 (83%)
Positive 498 (17.1%) 122 (16.6%) 243 (17.2%) 255 (17%)

Most extensive primary surgery 0.473
Mastectomy, NOS 1458 (50%) 378 (51.5%) 734 (52%) 724 (48.2%)
Partial mastectomy / lumpectomy / excisional biopsy 1457 (50%) 356 (48.5%) 678 (48%) 779 (51.8%)

PerioperativeAdjuvant Chemotherapy <0.001
Missing 0 (0%) 1 (0.1%) 0 (0%) 0 (0%)
No 310 (10.6%) 81 (11%) 142 (10.1%) 168 (11.2%)
Yes - Any neoadjuvant 575 (19.7%) 193 (26.3%) 267 (18.9%) 308 (20.5%)
Yes - Postoperative only 2030 (69.6%) 459 (62.5%) 1003 (71%) 1027 (68.3%)

PerioperativeAdjuvant Hormone Therapy 0.003
No 1087 (37.3%) 322 (43.9%) 527 (37.3%) 560 (37.2%)
Yes – Any neoadjuvant 7 (0.2%) 1 (0.1%) 3 (0.2%) 4 (0.3%)
Yes – Postoperative only 1821 (62.5%) 411 (56%) 882 (62.5%) 939 (62.4%)

PerioperativeAdjuvant Trastuzumab 0.799
No 2411 (82.7%) 610 (83.1%) 1167 (82.6%) 1244 (82.8%)
Yes 504 (17.3%) 124 (16.9%) 245 (17.4%) 259 (17.2%)

Sample for rs11212617 SNP <0.001
Available 2747 (94.2%) 579 (78.9%) 1329 (94.1%) 1418 (94.3%)
Unavailable 168 (5.8%) 155 (21.1%) 83 (5.9%) 85 (5.7%)

rs11212617 SNP 0.396
AA 823 (30%) 179 (30.9%) 401 (30.2%) 422 (29.8%)
CA 1347 (49%) 267 (46.1%) 657 (49.4%) 690 (48.7%)
CC 577 (21%) 133 (23%) 271 (20.4%) 306 (21.6%)

Recruitment	The CCTG MA.32 Clinical Trial (Clinical Trials.gov identifier: NCT01101438; http://clinicaltrials.gov/show/NCT01101438, first posted April 12, 2010) is a Phase III, randomized trial that enrolled 3649 non-diabetic subjects between 2010 and 2013; subjects received standard surgical, chemotherapeutic (completed at least one month prior to enrollment), hormonal, biologic and radiation therapy for a T1-3, N0-3, M0 BC diagnosed during the previous year. Subjects with T1c N0 BC were eligible if they had at least one of: histologic grade III, lymphovascular invasion, negative estrogen (ER) and progesterone (PgR) receptors, HER2 positivity, Oncotype Recurrence Score ≥ 25 or Ki-67 over 14%. In May 2012, after 2382 subjects were enrolled, eligibility criteria were amended to mandate triple negative (ER negative, PgR negative, HER2 negative) status for patients with T1cN0 disease and at least one of the above adverse tumor characteristic for patients with T2N0 tumors. Participants were required to have a fasting glucose of 7.0 mmol/L or lower; those with a history of diabetes, lactic acidosis, current use of diabetes medication, breast cancer recurrence or previous invasive cancer, excessive alcohol intake, or marked hepatic, kidney or cardiac dysfunction were excluded.
Ethics oversight	The study protocol was approved by institutional review boards of participating institutions, including the NCI (US) Central Institutional Review Board and Mount Sinai Hospital (Ontario Cancer Research Ethics Board). All patients provided written informed consent to participate.

Note that full information on the approval of the study protocol must also be provided in the manuscript.

Clinical data

Policy information about clinical studies

All manuscripts should comply with the ICMJE guidelines for publication of clinical research and a completed CONSORT checklist must be included with all submissions.

Clinical trial registration	Clinical Trials.gov identifier: NCT01101438; http://clinicaltrials.gov/show/NCT01101438
Study protocol	NCT01101438; http://clinicaltrials.gov/show/NCT01101438 or at Canadian Cancer Trials Group (cctg.ca)
Data collection	Recruitment took place 2010-2013 at multiple centers in Canada, United States, United Kingdom and Switzerland.
Outcomes	Paired bloods (baseline, 6 months) were assayed (blinded to treatment allocation) in batches with 10% random repeats for insulin (Roche ElectroChemiLuminescence Immunoassay (ECLIA)), leptin (Luminex Milliplex MAP assay) and hsCRP (Roche, particle based immunoturbidimetric assay). Blood was analyzed in 2014 -2015. Intra-assay coefficients of variability were 3%, 3% and 4% for insulin, leptin and hsCRP respectively. Glucose was analyzed at local clinical laboratories immediately after collection. HOMA (a marker of insulin resistance) was calculated from glucose and insulin levelssubjects when both were measured on the same day [glucose (mg/dl) x insulin (pmol/L) /22.5].16. One aliquot was sent on dry ice for genomic DNA extraction and genotyping for the SNP rs11212617 (Chr11(GRCh38):g.108412434C>A).
